# Topical naltrexone increases aquaporin 5 production in the lacrimal gland and restores tear production in diabetic rats

**DOI:** 10.3389/ebm.2024.10175

**Published:** 2024-05-02

**Authors:** David Diaz, Joseph P. Sassani, Ian S. Zagon, Patricia J. McLaughlin

**Affiliations:** ^1^ Department of Neural and Behavioral Science, Penn State University College of Medicine, Hershey, PA, United States; ^2^ Department of Ophthalmology, Penn State Health, Hershey, PA, United States

**Keywords:** lacrimal functional unit, lacrimal glands, naltrexone, type 1 diabetes, corneal surface

## Abstract

Diabetes mellitus is a prevalent disease that is often accompanied by ocular surface abnormalities including delayed epithelial wound healing and decreased corneal sensitivity. The impact of diabetes on the lacrimal functional unit (LFU) and the structures responsible for maintaining tear homeostasis, is not completely known. It has been shown that the Opioid Growth Factor Receptor (OGFr), and its ligand, Opioid Growth Factor (OGF), is dysregulated in the ocular surface of diabetic rats leading to overproduction of the inhibitory growth peptide OGF. The opioid antagonist naltrexone hydrochloride (NTX) blocks the OGF-OGFr pathway, and complete blockade following systemic or topical treatment with NTX restores the rate of re-epithelialization of corneal epithelial wounds, normalizes corneal sensitivity, and reverses dry eye in diabetic animal models. These effects occur rapidly and within days of initiating treatment. The present study was designed to understand mechanisms related to the fast reversal (<5 days) of dry eye by NTX in type 1 diabetes (T1D) by investigating dysregulation of the LFU. The approach involved examination of the morphology of the LFU before and after NTX treatment. Male and female adult Sprague-Dawley rats were rendered hyperglycemic with streptozotocin, and after 6 weeks rats were considered to be a T1D model. Rats received topical NTX twice daily to one eye for 10 days. During the period of treatment, tear production and corneal sensitivity were recorded. On day 11, animals were euthanized and orbital tissues including conjunctiva, eyelids, and lacrimal glands, were removed and processed for histologic examination including immunohistochemistry. Male and female T1D rats had significantly decreased tear production and corneal insensitivity, significantly decreased number and size of lacrimal gland acini, decreased expression of aquaporin-5 (AQP5) protein and decreased goblet cell size. Thus, 10 days of NTX treatment restored tear production and corneal sensitivity to normal values, increased AQP5 expression, and restored the surface area of goblet cells to normal. NTX had no effect on the number of lacrimal gland acini or the number of conjunctival goblet cells. In summary, blockade of the OGF-OGFr pathway with NTX reversed corneal and lacrimal gland complications and restored some components of tear homeostasis confirming the efficacy of topical NTX as a treatment for ocular defects in diabetes.

## Impact statement

In this study, our experiments confirmed that type 1 diabetes results in decreased tear fluid and decreased corneal sensitivity, with dysregulation in the lacrimal functional unit (i.e., decreases in lacrimal gland number and aquaporin 5 secretion, and number of conjunctival goblet cells). Topical NTX application restores tear production and corneal sensitivity, and increases the amount of aquaporin-5 expression in lacrimal gland acinar cells, but does not alter the defective morphology of the lacrimal functional unit suggesting that the rapid reversal of dry eye is related to corneal nerve sensitivity.

## Introduction

More than 38 million people in the United States are diagnosed with diabetes and another 38% of the adult population in 2023 has pre-diabetes [1]. There is an increased risk for this multifactorial disease in African Americans, Hispanics, and Pacific Islanders, as well as the overall aging community [1, 2]. Despite pharmaceutical control of diabetes, nearly 50% of persons will experience at least one complication related to tear production [3, 4]. Dry eye disease (DED) is characterized as an inflammatory disorder of unknown origin, with a multifactorial etiology, which presents with persistent low tear tear-film production, increased frequency of corneal ulcerations, irritated corneal surface, and altered mucin expression [3–5]. Diabetes is also accompanied by ocular surface disease including delayed epithelial wound healing and decreased corneal sensitivity [6–8]. Previous research in the laboratory has reported that type 1 diabetic (T1D) rats have elevated serum and corneal tissue levels of this inhibitory neuropeptide called Opioid Growth Factor (OGF) [7]. Chemically termed methionine-enkephalin, OGF also has been shown to be elevated in humans with diabetes [9, 10]. Our studies have documented that OGF binds to the nuclear-associated receptor OGFr which results in reduced cell replication in the corneal surface [11]. Blockade of these effects using the opioid antagonist NTX either systemically or topically results in reversing many of the epithelial associated complications of type 1 or type 2 diabetes including decreased corneal surface sensitivity, delayed corneal wound healing, and dry eye [12–14]. Recent investigations in our laboratory have reported the dysregulation of the OGF-OGFr axis corresponds with the onset and severity of dry eye in both male and female hyperglycemic rats [7, 15]. Twice daily topical administration of NTX for 6 weeks reversed the dry eye and restored the homeostasis of the corneal surface in these T1D animals [16].

In order to begin assessing the mechanisms related to NTX-related reversal of reduced tear production in diabetes, we investigated the morphology of the lacrimal functional unit (LFU) in untreated diabetic rats and in diabetic animals treated with NTX. Tear production results from secretions of the LFU, first defined in 2007 by International Dry Eye Workshop to include lacrimal glands, conjunctiva, corneal surface, Meibomian glands, and eyelids as well as the vasculature and nervous system connections [17] are explored by McKown et al. [18]. Knowledge about the morphological status of the lacrimal gland and its function in the secretion of ocular mucins is important in understanding DED [19, 20]. The different roles of the lacrimal gland in rodent versus human DED are still debated [21], but there is considerable agreement regarding the importance of inflammation and other aspects of the pathophysiology of the lacrimal gland to deciphering the causes and treatments of DED [18, 19]. Information on the LFU status in hyperglycemia and/or diabetes is sparse but researchers have shown that hyperglycemia induces dry eye through alterations in the LFU, as well as autonomic neural pathways, particularly the norepinephrine-adrenergic receptor–mitochondrial pathway [22]. The specific role of the OGF-OGFr axis in inducing or perpetuating tear flow is unknown. Nevertheless, blockade of the OGF-OGFr axis has an immediate impact on dry eye associated with diabetes, and as this study demonstrates, topical NTX treatment can reverse complications such as dry eye is within days. Our *hypothesis* is that the morphology of the LFU, specifically conjunctival goblet cells, and lacrimal gland acini, are dysregulated in size and function in diabetes and contribute to dry eye and corneal insensitivity. Moreover, we postulate that treatment with NTX will reverse histopathologic and immunohistochemical abnormalities induced in the lacrimal gland in diabetes, which eventuate in dry eye.

## Materials and methods

### Animals and treatment

The study design was approved by the Penn State College of Medicine Institutional Animal Use Committee (IACUC) and followed regulations stated by the ARVO guideline for Use of Animals in Ophthalmic and Vision Research.

Male and female rats weighing approximately 150 g and 140 g, respectively, were purchased from Charles River Laboratories, Wilmington MA and housed in standard animal facilities receiving 12-12 h light-dark cycle, 14-18 changes of air hourly, and controlled humidity and temperature. Rats were placed 2 per cage and food and water were available *ad libitum*.

Hyperglycemia was induced as previously reported [13–15]. Briefly, animals were fasted for 4 h and then given an intraperitoneal injection with 50 mg/kg streptozotocin (STZ; Sigma-Aldrich) dissolved in citrate buffer, pH 4.5, on two consecutive days. Tail vein glucose was measured using a glucometer after 72 h; readings greater than 300 mg/dL were designated as hyperglycemic. At this time, rats were arranged such that hyperglycemic rats of the same sex were housed together in order to accommodate extra cage changes required by the increased urination. Animals that did not become hyperglycemic within 5 days of STZ injection were removed from the study.

After more than 6 weeks of hyperglycemia, animals were designated as type 1 diabetic (T1D) [23] and randomly assigned to a cohort treated with topical NTX drops (50 µL) twice daily for 10 days; these animals were designated T1D-NTX. A single drop containing 5 × 10^-5^ M NTX was administered without anesthesia to the right eye only at 8–9 a.m. and 5–6 p.m. daily. T1D rats receiving vehicle-containing drops were designated as T1D and non-diabetic normal rats as Normal, were administered 50 µL of vehicle within the same time period. Male and female rats were treated comparably.

For comparison and to insure that the effects of NTX were not harmful and related to diabetes, a separate cohort of Normal animals received NTX drops (Normal-NTX) or vehicle for 10 days. Tear volume was measured at baseline and at 5 and 10 days after drops.

To confirm rigor and reproducibility, four independent experiments using male rats and three independent experiments with female rats were conducted in a sequential manner. Ocular tissues from 12 diabetic males, 14 diabetic females, 8 non-diabetic normal males, and 9 non-diabetic Normal females were evaluated.

### Ocular surface complications: *in vivo* measurements

Tear production and corneal sensitivity were measured in both male and female rats after 6 weeks of hyperglycemia. Measurements were made before initiating the NTX treatment (time 0) and at 5 and 10 days of NTX treatment; recordings at 5 and 10 days were taken prior to the morning treatment. All measurements were recorded on unanesthetized animals following published procedures [13–15].

#### Schirmer tests

Briefly, tear production was measured using Schirmer strips cut at 1 mm × 17 mm length and placed into the lower lid cul-de-sac for 60 s. The wetting distance was measured to the half-millimeter using the manufacturer’s scale.

#### Corneal surface sensitivity

Corneal surface sensitivity was measured using the blink reflex and the Cochet-Bonnet aesthesiometer (Boca Raton, FL). The amount of force (g/mm^2^) required to induce blinking was considered indicative of corneal sensitivity with greater pressure values indicative of insensitivity. Decreasing lengths of the filament were touched to the center of the cornea to elicit a blink reflex. The length of the filament required to induce a blink reflex was recorded; the procedure was repeated 3 times and averaged. The length was then converted to g/mm^2^ using the manufacturers provided conversion chart.

### Morphological changes in the LFU in diabetic rats

One day after NTX treatment was concluded, all rats in the study were humanely euthanized using a Euthanex carbon dioxide apparatus followed by decapitation. Orbital tissues were excised including the globes, eyelids containing Meibomian glands, conjunctiva, Harderian glands and external lacrimal glands. Tissues were fixed either in 4% paraformaldehyde for 1 h at 20°C followed by 12 h in 30% sucrose at 2–8°C or fixed in Hartman’s Fixative fir 12h at 20 °C and embedded in paraffin. Fresh-frozen tissues were frozen in chilled isopentane and stored at −80°C until cryosectioned. Tissues were stored for less than 10 days before processing for immunohistochemistry.

#### LFU morphology

Overall LFU morphology was assessed in paraffin-embedded hematoxylin and eosin (H&E) or periodic acid Schiff (PAS) stained sections (6-8um) of lacrimal glands and eyelids containing Meibomian glands and goblet cells. Goblet cell number and size were assessed in PAS-stained conjunctival tissues measuring 200um of conjunctiva starting at the deepest portion of the conjunctival crypt; goblet cells below 150 μm^2^ were excluded from counts. The number and size of acini in the Meibomian and lacrimal glands were measured in PAS-stained sections. Briefly, acini were counted from stained images that were imported into ImageJ software. Original images were photographed at ×40 magnification on an Olympus BX50 microscope. The number of acini (≥24) within defined areas (200 µm in diameter) was counted using at least 3 sections per animal and 3 animals per group for each sex.

#### Immunohistochemical (IHC) staining

Frozen sections (14–16 µm) of lacrimal glands were stained with validated antibodies for aquaporin-5 (AQP5; 1:100; ThermoFisher) followed by secondary antibodies using Alexafluor 568 (1:1000; ThermoFisher). IHC staining was assessed using confocal microscopy on an Olympus IX81 with Fluoview (FV1000) at ×40 magnification.

### Data analysis

IHC staining was measured using optical density. ImageJ software was calibrated as indicated by ImageJ protocols. Mean intensity of samples was normalized to the number of DAPI positive cells in the area captured. Data from the independent experiments were combined for each sex and analyzed using ANOVA (two-way or three-way) with a Tukey *post hoc* test for multiple comparisons. Throughout the study, data are expressed as SEM. All analyses were performed using GraphPad Prism version 9.0 (GraphPad Software, Inc.); *p* < 0.05 was considered statistically significant.

## Results

### Clinical signs

Male rats weighed approximately 146 ± 9 g at the time of inducing hyperglycemia. Eight weeks later Normal male rats weighed 493 ± 52 g and T1D animals weighed significantly less (*p* < 0.01) than Normals (approximately 328 ± 45 g). Female rats weighed 143 ± 6 g prior to inducing hyperglycemia, and following STZ injections at 8 weeks weighed 262 ± 10 g and 224 ± 5 g (*p* < 0.01) for Normal and T1D rats, respectively. Blood glucose levels at 72 h after STZ were approximately 480 mg/dL for rats injected with STZ (i.e., hyperglycemic) and for the non-injected Normal rats, glucose levels were ∼120 mg/dL. NTX eyedrops had no effect on body weight or blood glucose levels in both sexes; data corroborated previous studies in this regard [7, 15, 16].

### Mean tear volume and corneal sensitivity

**FIGURE 1:**
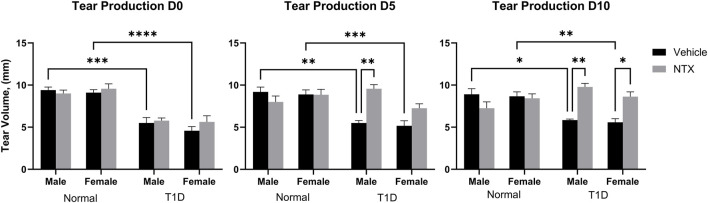
Tear production (volume, µm) in male **(A)** and female **(B)** Sprague-Dawley rats randomly selected from Normal, T1D, T1D-NTX, and Normal-NTX groups (N ≥ 6) at each time point. Values represent means ± S.E.M as measured by the Schirmer test. Data were analyzed using three-factor ANOVA (condition, treatment and sex) with *post hoc* Tukey’s tests. Significant differences on each day were noted as *p* < 0.05 (*), *p* < 0.01 (**), *p* < 0.001 (***), or *p* < 0.0001 (****).

After 6–7 weeks of hyperglycemia, tear production measurements as indicated by the length of wetting of the Schirmer strip were taken at baseline and after 5 and 10 days of NTX treatment for both males and females in all Normal, T1D, and treated cohorts; values are presented in Figure 1. Three-factor ANOVAs between condition (T1D, normal), treatment (NTX, vehicle), and sex were performed. At time 0, there were no interactions involving treatment, but both male and female T1D mice had Schirmer scores that were significantly less than respective Normal rats of corresponding sex. Baseline Schirmer scores for male T1D rats were 5.5 ± 0.6 mm, a significant (*p* < 0.001) reduction of approximately 42% from normal values of 9.7 ± 0.6 mm. Baseline Schirmer scores for female T1D rats were 5.625, 38.26% less than Normal females (*p* < 0.0001). Three-factor ANOVA performed on day 5 showcased a significant interaction within condition (F (1, 47) = 18.38; *p* < 0.0001), treatment (F (1, 47) = 8.008; *p* = 0.0068) and between Condition x treatment (F (1, 47) = 18.03; *p* = 0.0001). Within 5 days of NTX treatment, T1D male rats receiving NTX had substantially improved Schirmer scores that were significantly elevated above T1D rats receiving vehicle (*p* < 0.01) and comparable to Normal male rat values. Female T1D rats receiving NTX improved but did not reach significant levels in their Schirmer scores. After 10 days of NTX treatment, both male and female T1D rats receiving NTX had significantly higher (*p* < 0.01 for males; *p* < 0.05 for females) Schirmer scores that did not differ from Normal sex counterparts. T1D rats receiving vehicle continued to display reduced Schirmer values.

**FIGURE 2:**
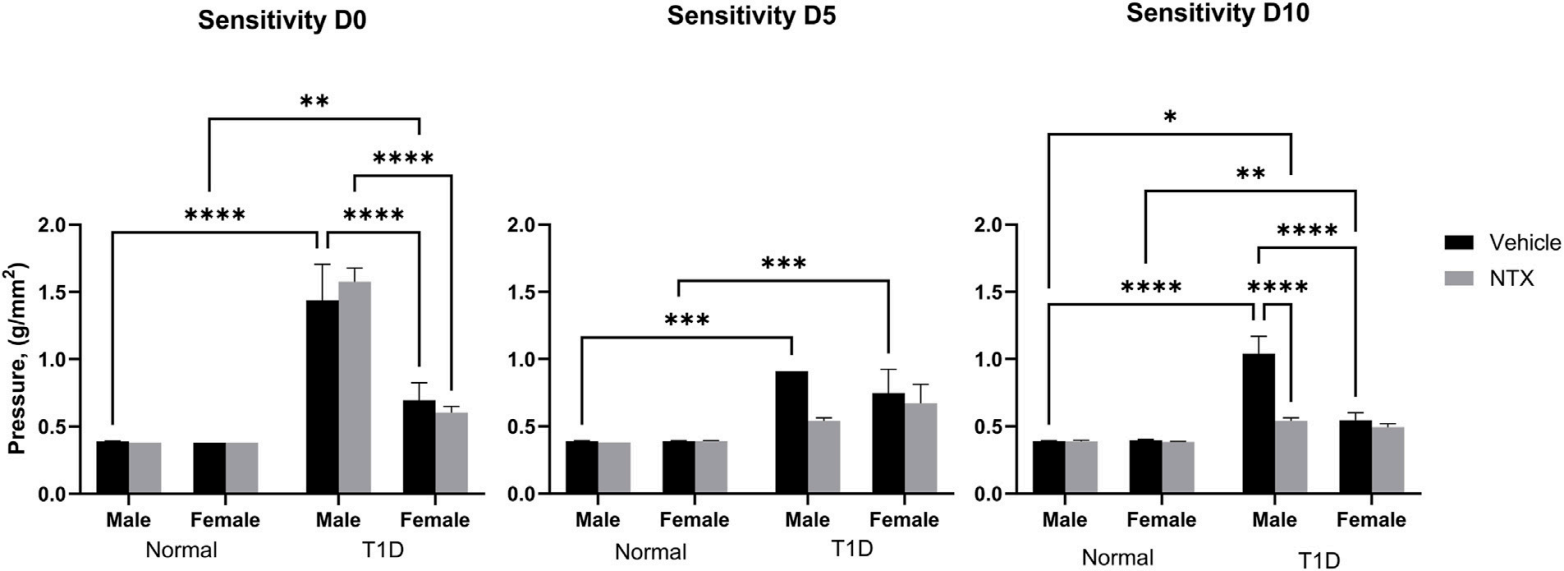
Corneal surface sensitivity measured with a Cochet-Bonnet aesthesiometer in male **(A)** and female **(B)** Sprague-Dawley rats. Three readings of pressure (g/mm^2^) were averaged for each rat at every time point. test. Values represent means ± S.E.M. for rats randomly selected at each time point (N ≥ 6 per group). Data were analyzed using three-way ANOVA (condition, treatment, sex) with *post hoc* Tukey’s tests. Significant differences on each day were noted as *p* < 0.05 (*), *p* < 0.01 (**), *p* < 0.001 (***) or *p* < 0.0001 (****).

*Corneal sensitivity* was analyzed with a three-factor ANOVA (Figure 2). At baseline (time 0), Normal male and female rats had comparable pressure values of 0.39 g/mm force. T1D male rats required significantly (*p* < 0.0001) greater force to elicit a blink response. Female T1D rats at baseline required 0.65 g/mm force to elicit a blink; these values differed from both male T1D and Normal female rats. After 5 days of NTX treatment, male and female T1D rats receiving NTX had sensitivity scores comparable to Normals, but did not differ substantially from T1D rats receiving vehicle. After 10 days of NTX treatment, T1D male and female rats continued to have depressed sensitivity (i.e., elevated pressure scores). However, NTX treatment stabilized sensitivity in male T1D rats such that these animals had sensitivity scores that were significantly less (*p* < 0.0001) than T1D counterparts receiving vehicle.

### Morphology of the lacrimal functional unit

#### External lacrimal gland

**FIGURE 3:**
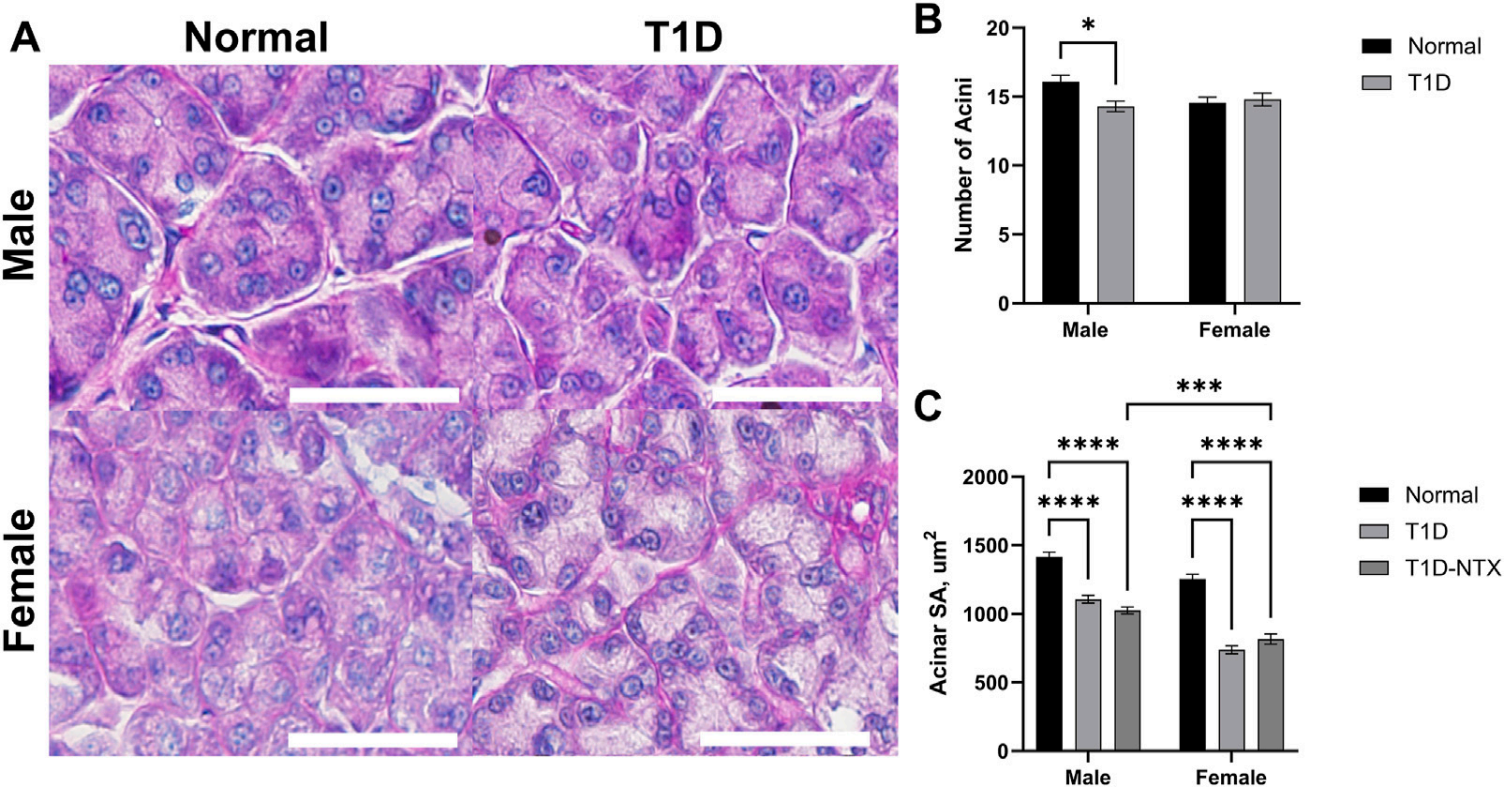
Morphology of lacrimal glands in male and female Sprague-Dawley rats. **(A)** Morphological images of lacrimal glands showing acini in male and female Normal and T1D rats. Tissues were stained with hematoxylin and eosin and photographed using an Olympus BX50 microscope at ×40 magnification. Bar = 50 µm. **(B)** Histograms representing the number of acini (mean ± SEM). **(C)** The mean area of individual acini (µm^2^) for males and females in each cohort are recorded from areal measurements for >60 acini taken from images of at least 6 lacrimal glands per group for each sex. Data were analyzed with multiple factor ANOVA. There was a significant interaction between sex and treatment. Significantly different at *p* < 0.05 (*), *p* < 0.001 (***) and *p* < 0.0001 (****).

The number, size, and shape of lacrimal gland acini in PAS-stained sections are presented in Figure 3 for male and female Normal and diabetic rats. As captured in the images (Figure 3A), male diabetic rats had fewer (*p* < 0.05; n = 14 acini per 100 μm^2^) acini per lacrimal gland (Figure 3B) that were significantly smaller (*p* < 0.0001) in area than those in Normal male rats (Figure 3C). Regarding surface area of acini for male rats, Normal animals had mean areas of 1,415 ± 33 μm^2^ in comparison to T1D rats with mean acini areas of 1,106 ± 27 μm^2^. Ten days of NTX treatment did not alter the number (data not shown) or size of acini. Two-factor ANOVA revealed a significant interaction between sex vs. treatment (F (2, 444) = 5.215; *p* = 0.0058) followed by *post hoc* comparisons that reveals the acinar size in female T1D-NTX rats was significantly smaller than for male T1D-NTX rats (*p* < 0.001). No differences in the number of acini were recorded for female rats in the Normal or T1D cohorts (Figure 3B), but the average surface area for lacrimal glands was smaller (Figure 3C) in T1D and T1D-NTX female rats relative to Normal females. Similar to males, 10 days of NTX treatment had no affect on the morphology of acini from female diabetic rats.

**FIGURE 4:**
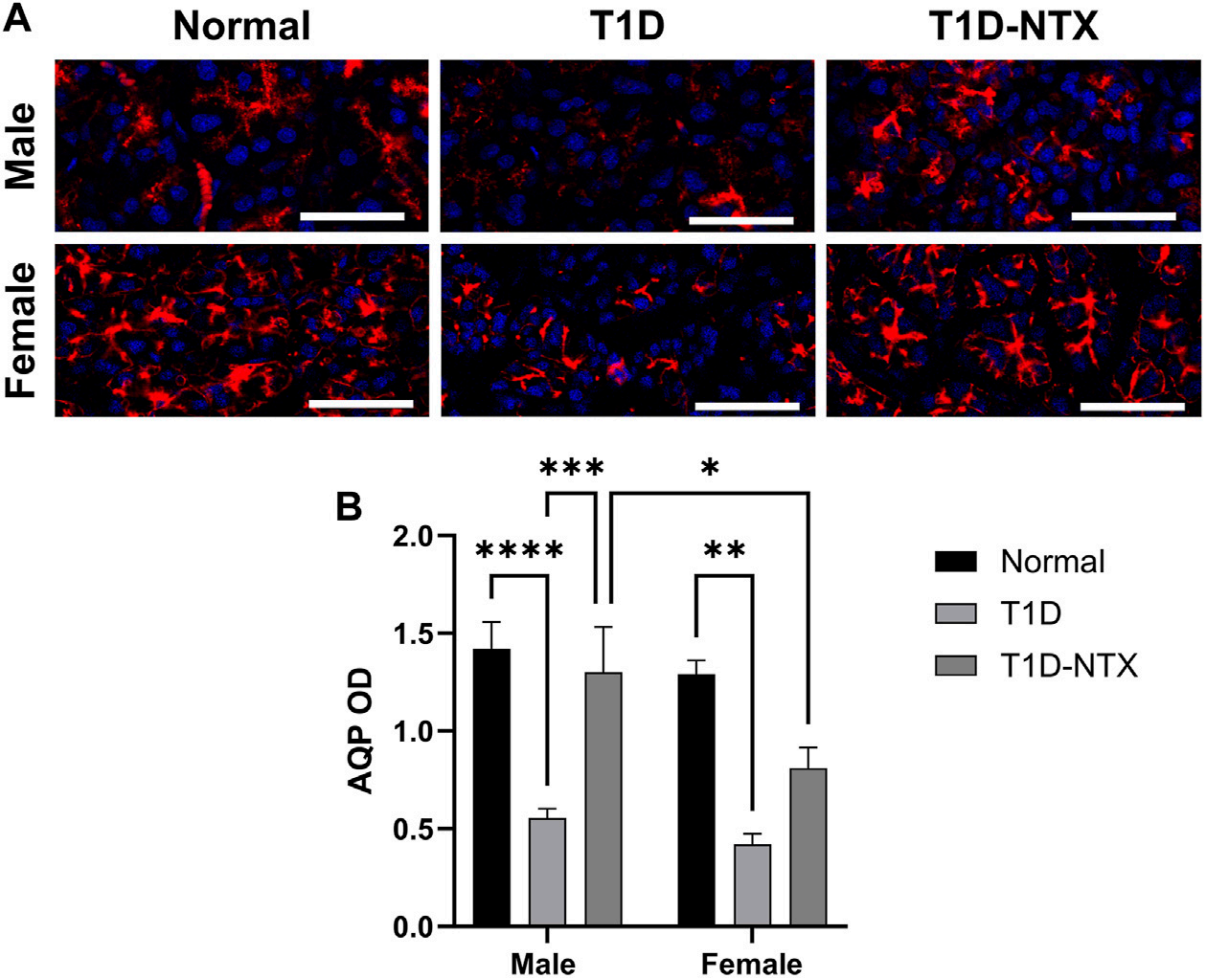
Aquaporin 5 expression in lacrimal glands of male and female normal and T1D rats. **(A)** Immunohistochemical images of aquaporin 5 expression in lacrimal glands of male and female Normal, T1D, and T1D-NTX rats. Tissues were stained with anti-aquaporin 5 (1:100) and AlexaFluor568 (1:1000) and images captured using Olympus IX81 confocal microscopy at ×40 magnification. Bar = 50 µm. **(B)** Histogram values represent means ± SEM for optical density measurements. Two-way ANOVA showed no significant interaction between sex and treatment. Primary factors were significantly different at *p* < 0.05 (*), *p* < 0.01 (**), *p* < 0.001 (***), and *p* < 0.0001 (****).

The function of lacrimal gland acini is to produce and release fluid in order to lubricate, protect, and cleanse the corneal surface. One of the major proteins in the aqueous tear film is aquaporin-5. Reduced levels of tear film aquaporin-5 are associated with dry eye and/or other epithelial surface pathologies [24]. Aquaporins aid in transporting water across cell membranes. Aquaporin-5 is located in salivary and lacrimal glands, as well as corneal epithelium and alveolar type 1 cells. In the current experiments, AQP5 was measured by optical density in tissue (Figure 4A). Optical density measurements supported the visual images of lacrimal glands. Values were significantly decreased in stained sections from male (*p* < 0.0001) and female (*p* < 0.01) T1D rats relative to sex-matched Normals (Figure 4B). Within 10 days of topical NTX application, aquaporin secretion was substantially increased (*p* < 0.001) in T1D-NTX male rats, but not in female T1D-NTX rats. A two-factor ANOVA did not show a significant interaction between sex and treatment, but did reveal that treatment had a significant effect (F (2, 66) = 17.02; *p* < 0.0001) on AQP5 presence in lacrimal gland tissue. Post hoc comparisons showed female T1D-NTX rats differed significantly from T1D-NTX male rats at *p* < 0.05 and had comparable AQP5 values as T1D female rats.

#### Size and number of conjunctival goblet cells

**FIGURE 5:**
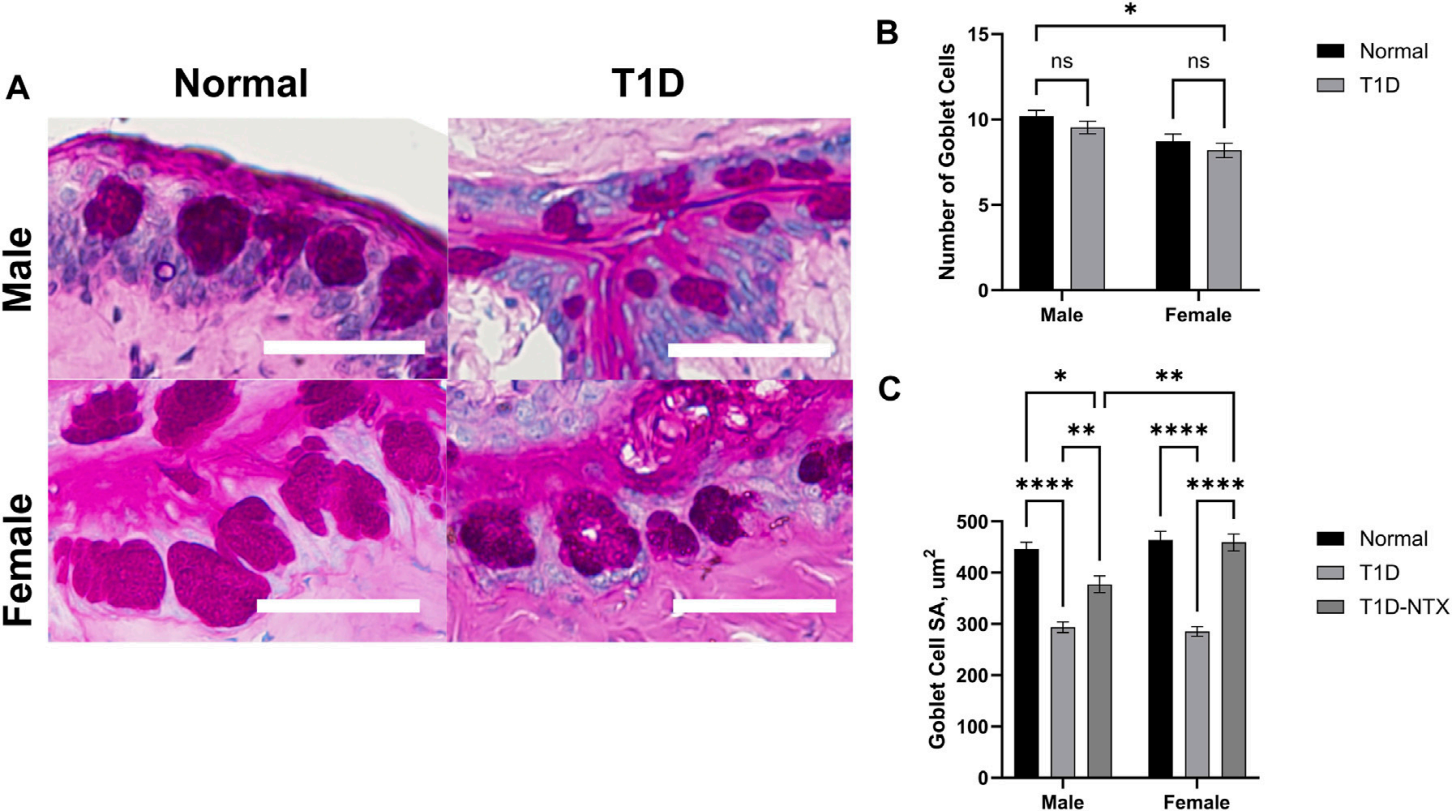
Analysis of goblet cells in the conjunctiva of male and female Normal and T1D rats. **(A)** Morphological images of the conjunctiva showing PAS-stained goblet cells. Images were taken using an Olympus BX50 microscope at ×40 magnification. Bar = 50 µm. **(B)** Histograms of the number (mean ± SEM) of goblet cells in Normal and T1D rats. **(C)** Histograms of (means ± SEM) of the surface area (µm^2^) of individual goblet cells. Analyses of variance indicated no significant interaction between sex and treatment for the number of goblet cells, but a significant interaction between sex and treatment (*p* = 0.006) for area. Post-hoc analyses showed differences at *p* < 0.05 (*), *p* < 0.01 (**), and *p* < 0.0001 (****).

Goblet cells produce and secrete mucins that are an important component of the precorneal tear film by helping it to adhere to the ocular surface [20]. The mucin also is a repository for immunoregulation [22]. Goblet cells in the conjunctiva were stained with hematoxylin and eosin and exhibited changes in the surface area in T1D rats in comparison to Normal animals of the same sex (Figures 5A–C). Goblet cell number was measured 200 µm from the deepest part of the conjunctival crypts, it was comparable between Normal and T1D rats of both sexes (Figure 5B). The surface area (Figure 5C) calculated from measurements of 65–100 cells in each treatment cohort revealed that the stained surface secretions in both male and female T1D rats were approximately 293.5 ± 10 μm^2^ whereas both sexes of Normal rats had cells with mean surface areas of approximately 446.1 ± 14 μm^2^ (*p* < 0.0001). NTX treatment for 10 days increased the surface area for both male (*p* < 0.01) and female (*p* < 0.0001) T1D-NTX rats. A significant two-way interaction between sex and treatment was noted (*p* = 0.0065), followed by *post hoc* comparisons which revealed that female rats treated with NTX had significantly more mucin (*p* < 0.01) than male T1D-NTX rats.

#### Meibomian surface area

The surface area of the Meibomian glands, a representation of size and secretory capacity, were significantly smaller in both male T1D and female T1D rats relative to Normals (data not shown). The mean surface areas for Normal males and females were 4,884 ± 334 μm^2^ and 5,167 ± 229 μm^2^, respectively with approximately a 27% decrease in size in both male and female T1D glands. There were marginal increases in surface recorded in female T1D receiving 10 days of NTX, with no increase in area in male T1D rats.

## Discussion

This study analyzed morphological and functional alterations in the LFU of diabetic rats treated with topical NTX. The LFU is essential in producing and maintaining a healthy tear film to help preserve ocular surface homeostasis. Previous studies have shown that diabetes produced altered morphology in the LFU structures, with decreased secretory granules in the lacrimal gland, decreased conjunctival goblet cell density, and decreased acinar size in Meibomian glands [25–27]. We found morphological alterations in the diabetic LFU after 6–7 weeks of hyperglycemia, with topical NTX altering only goblet cell deficits after 10 days of treatment. Topical treatment increased AQP5 expression levels in the lacrimal gland which we associated with increased tear secretion.

Conjunctival goblet cells aid in the production of the mucin layer of the tear film, which are responsible for providing a protective barrier against pathogens and providing lubrication [28]. Mucins have altered production and expression in dry eye syndrome with studies using a MUC5AC knock out mouse model to simulate dry eye disease in animals [29]. Our results revealed that topical NTX caused a restoration in the size of the conjunctival goblet cell and was interpreted as an increased production of cellular projects.

Similarly, diabetic Meibomian glands also showcased a decrease in acinar size. The Meibomian glands are responsible for the lipid layer of the tear film, which studies have shown to be dysregulated in persons with diabetes [30]. The lipid layer prevents desiccation and is described in the pathology of desiccative dry eye disease. Both the mucin and lipid layers of the tear film would not be directly responsible for the reflexive tear secretion produced by the Schirmer strips. This is a response caused by afferent corneal nerves being stimulated, causing parasympathetic activation of the lacrimal gland to “flush” irritants out of the corneal surface.

Our results support previous studies showcasing atrophied acini in the diabetic lacrimal gland that may reflect decreased cell size and/or secretory function [24, 31]. Studies have shown that diabetic rats have smaller secretory vesicles, decreased levels of cations, and decreased total protein [24, 32]. Topical naltrexone treatment did not alter the morphology of the lacrimal gland after 10 days. To investigate if there was an alteration in the secretory function of the lacrimal gland, we stained for AQP5. AQP5 is a water channel protein that plays a role in the generation of saliva, pulmonary secretions, and tears. Reduced expression of AQP5 has been measured in patients with Sjögren syndrome, and autoimmune disorder which commonly presents with dryness in the eyes and mouth [33]. Treatment with topical NTX increased expression levels of AQP5 in the lacrimal gland, which we attribute to stimulation by autonomic nerves causing the corneal reflex. Topical naltrexone may be modulating corneal nerve sensitivity or promoting nerve growth to allow for normal signaling. Topical analgesic drugs used in another study, showcased a similar increase in tear production and increase in AQP5 within the lacrimal gland, suggesting that corneal nerves can be affected by topical treatment [34].

Corneal stimulation activates polymodal nociceptors which trigger autonomic fibers responsible for lacrimal gland secretion. The cornea is the most densely innervated and sensitive tissue in the body [35]. Peripheral neuropathy is common complication in diabetics, described as nerve damage that preferentially targets sensory axons, it can also occur in the cornea causing a decrease in corneal nerve density, decreased wound healing, and has been associated with dry eye disease [36–38]. Studies have suggested that corneal nerve density can increase within 1–2 weeks of topical treatment, with increases in corneal would healing [39, 40]. We have previously shown that topical naltrexone increased corneal wound healing and increased corneal sensitivity, which we believe could be due to nerve regeneration or the release of neurotrophic factors by the corneal epithelial cells [13].

Type-1 diabetes in rats resulted in decreased LFU size, showcasing smaller acini in the meibomian and lacrimal glands, and reduced goblet cells within the conjunctival crypts. Ten-day topical NTX treatment resulted with increased goblet cell size, but did not show any changes to meibomian and lacrimal gland morphology. The increased tear production was attributed to increased secretory function within the lacrimal gland, inferred by the increased AQP5 staining expression. We suspect the reduced size in LFU structures is caused by decreased secretory function, which may be due to reduced signaling by autonomic pathways. NTX binds on corneal epithelial cells and blocks OGFr from binding to its ligand, OGF. Blockage of this negative growth pathway may result in decreased corneal regeneration, causing a poor signaling from corneal afferent nerves. Alternatively, naltrexone could indirectly promote the release of neurotrophic factors by altering corneal epithelium responsible for their production, creating interactions where denervation alters corneal epithelium and corneal epithelium can influence the survival of corneal nerves [41, 42]. Topical naltrexone does not seem to be affecting the LFU directly, instead it may be causing alterations to corneal nerve signaling pathways to cause downstream increased tear production.

## Conclusion

In conclusion, our results indicate that type-1 diabetes alters the LFU morphology, and decreases lacrimal gland secretory function. The data clearly show increased tear production and AQP5 expression after NTX treatment. Future studies will investigate corneal nerve density in naltrexone treated diabetic rats. Naltrexone can block the growth inhibitor effects of a dysregulated OGF-OGFr axis which could be involved with delayed nerve growth in diabetic corneas. By looking at neurotrophic abundance or corneal nerve density we hope to elucidate the mechanism of naltrexone in dry eye.

## Data Availability

The raw data supporting the conclusion of this article will be made available by the authors, without undue reservation.
